# Mogamulizumab‐Associated Autoimmune Diseases: Insights From FAERS Database Analysis

**DOI:** 10.1002/cam4.70478

**Published:** 2024-12-10

**Authors:** Genshan Zhang, Haokun Zhang, Jie Fu, Zhixin Cao

**Affiliations:** ^1^ Department of Gastrointestinal Surgery, Tongji Hospital, Tongji Medical College Huazhong University of Science and Technology Wuhan People's Republic of China; ^2^ School of Public Health and Health Management Gannan Medical University Ganzhou People's Republic of China; ^3^ Department of Nursing, Tongji Hospital, Tongji Medical College Huazhong University of Science and Technology Wuhan People's Republic of China

**Keywords:** adverse events, disproportionality analyses, Food and Drug Administration Adverse Event Reporting System, mogamulizumab

## Abstract

**Background:**

Mogamulizumab is a monoclonal antibody targeting the C‐C chemokine receptor 4, used to treat T‐cell malignancies such as cutaneous T‐cell lymphoma, adult T‐cell leukemia/lymphoma, and peripheral T‐cell lymphoma. However, real‐world studies on mogamulizumab‐associated adverse events (AEs) are limited.

**Methods:**

Disproportionality analyses were performed to assess the safety profile of mogamulizumab based on data from the US Food and Drug Administration Adverse Event Reporting System (FAERS) database for the period spanning from October 2018 to December 2023. The research investigated demographic characteristics, the onset timing of AEs, and the safety implications associated with mogamulizumab use.

**Results:**

A total of 1182 significant preferred terms were identified among the 3661 mogamulizumab‐associated AE reports collected from the FAERS database. The frequently reported AEs including rash, infusion‐related reaction, and pyrexia were in line with drug instruction. Notably, several unexpectedly significant AEs were also found, including pemphigoid (ROR = 5.69 [95% CI 1.83–17.66]), unstable angina (ROR = 20.56 [95% CI 8.54–49.5]), bulbar palsy (ROR = 238.36 [95% CI 75.22–755.31]), myositis (ROR = 12.65 [95% CI 5.67–28.19]), and various autoimmune diseases such as autoimmune hepatitis (ROR = 21.33 [95% CI 11.08–41.07]), myocarditis (ROR = 15.29 [95% CI 8.67–26.97]), glomerulonephritis (ROR = 22.49 [95% CI 7.24–69.9]), nephrotic syndrome (ROR = 7.63 [95% CI 2.46–23.67]), myasthenia gravis (ROR = 8.54 [95% CI 3.2–22.77]), and autoimmune thyroiditis (ROR = 11.81 [95% CI 3.8–36.68]).

**Conclusion:**

This study replicated previously identified AEs associated with mogamulizumab and uncovered additional signals of AEs, particularly emphasizing the risks associated with autoimmune diseases. It is essential to exercise vigilance in monitoring the occurrence of these AEs during the use of mogamulizumab in clinical practice.

**Abbreviations**
ADCC: antibody‐dependent cell‐mediated cytotoxicity
AER: adverse event reports
AEs: adverse events
ATLL: adult T‐cell leukemia/lymphoma
BCPNN: Bayesian confidence propagation neural network
CCR4: chemokine receptor 4
CTCL: cutaneous T‐cell lymphoma
FAERS: Food and Drug Administration's adverse event reporting system
FDA: Food and Drug Administration
MedDRA: medical dictionary for regulatory activities
MGPS: multi‐item gamma Poisson shrinker
PRR: proportional reporting ratio
PTCL: peripheral T‐cell lymphoma
ROR: reporting odds ratio
SOC: system organ class
Treg: regulatory T

## 1 Introduction

The transmembrane chemokine receptor 4 (CCR4) is predominantly expressed on the surface of Th2 helper cells and regulatory T (Treg) cells [1, 2]. However, in T‐cell malignancies such as cutaneous T‐cell lymphoma (CTCL), adult T‐cell leukemia/lymphoma (ATLL), and peripheral T‐cell lymphoma (PTCL), the malignant T‐cell tumor cells often exhibit an overexpression of this receptor [2, 3]. Mogamulizumab is a monoclonal antibody that targets CCR4, promoting antibody‐dependent cell‐mediated cytotoxicity (ADCC) and leading to the elimination of target cells [4, 5, 6]. In a phase III clinical trial, mogamulizumab demonstrated a significant extension in patients' survival compared to vorinostat, unveiling a novel therapeutic avenue for CTCL [5]. Consequently, the U.S. Food and Drug Administration (FDA) authorized the utilization of mogamulizumab in adult patients grappling with relapsed/refractory mycosis fungoides, Sezary syndrome, and those who have previously undergone at least one systemic therapy [7]. Additionally, mogamulizumab is also used for treating other T‐cell lymphomas, including ATLL and PTCL [8, 9].

Although mogamulizumab exhibits promising clinical efficacy, it is also linked to various adverse reactions. Previous studies have shown that the most frequent adverse events (AEs) associated with mogamulizumab include upper respiratory tract infections, musculoskeletal pain, diarrhea, fatigue, infusion‐related reactions, and rash (reported incidence ≥ 20%) [7]. Approximately 36% of patients report experiencing serious AEs, with infections accounting for 16% of all patients [7]. In addition, refractory graft‐versus‐host disease, autoimmune complications, and skin toxicity have been observed as AEs [7, 10]. However, the safety data mainly come from clinical trials and postmarketing observational studies. Real‐world studies can compensate for the small sample size in clinical trials, yet research on the safety of mogamulizumab in the real world is limited.

The U.S. Food and Drug Administration's Adverse Event Reporting System (FAERS) provides abundant real‐world data that can be used to evaluate the safety and effectiveness of drugs [11]. This study aims to assess the safety of mogamulizumab after its approval through data mining of the FAERS database.

## 2 Materials and Methods

### 2.1 Data Sources

This study utilized ASCII report files from the FAERS spanning from the last quarter of 2018 to the last quarter of 2023 as the primary data source. Data management and visualization processing were conducted using MySQL 15.0 [12].

### 2.2 Data Extraction

**FIGURE 1:**
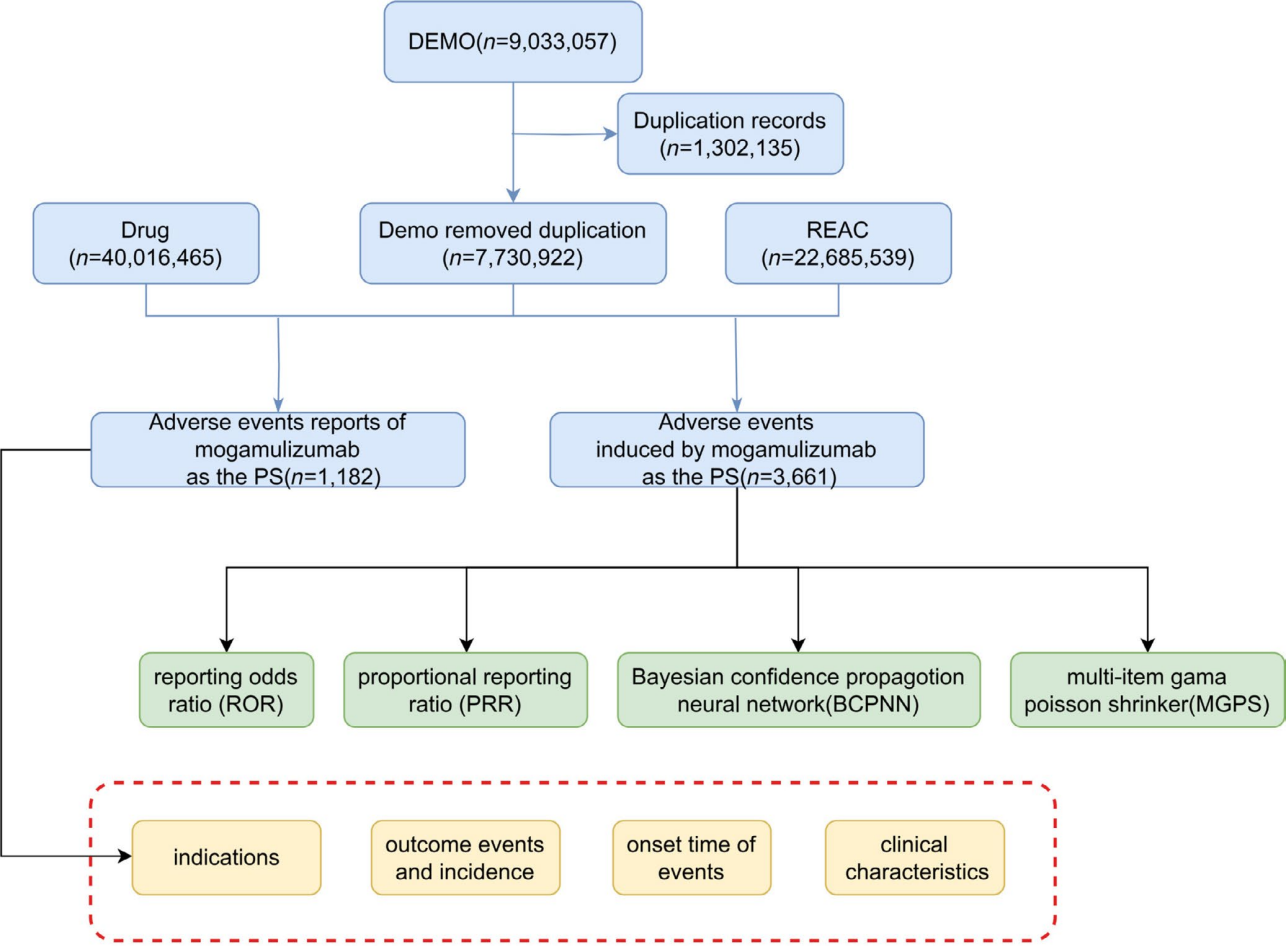
The process of selecting mogamulizumab‐associated AEs from FAERS database.

Duplicate and conflicting reports were eliminated from the files, retaining only the most recent case IDs to ensure data accuracy. The reports were identified using primary ID, and the drug names were standardized. Mogamulizumab was designated as the primary drug, and reports about AEs were collected for variable analysis, including patient age, gender, reporter types, and regions. The flowchart of data processing can be found in Figure 1.

### 2.3 Definition of AEs

During the preprocessing phase of this study, reports with preferred terms (PTs) < 3 were excluded. Medical Dictionary for Regulatory Activities (MedDRA) was employed to reposition, categorize, and encode AEs based on PT and system organ class (SOC) to analyze their impact on organ systems.

### 2.4 Statistical Analysis

Disproportionality analysis was used to determine the potential association between mogamulizumab and AEs, aiming to evaluate the correlation between the drug and AEs through comparing the ratio of observed frequencies in exposed and unexposed populations (Table S1). In this study, four disproportionality methods were employed to identify signals of AEs: reporting odds ratio (ROR) [13], proportional reporting ratio (PRR) [14], multiple‐item shrinker of Bayesian confidence propagation neural network (BCPNN) [14], and multi‐item gamma Poisson shrinker (MGPS) [15]. ROR offers the advantage of correcting biases induced by low numbers of event reports, while PRR demonstrates higher specificity in comparison to ROR. BCPNN excels in integrating diverse data sources and conducting cross‐validation. MGPS is particularly adept at detecting signals arising from rare events. The specific formulas and thresholds for these four methods can be found in Table S2. Higher values in this analysis indicate stronger signal strength, reflecting a more robust relationship between the target drug and AEs.

## 3 Results

### 3.1 Basic Characteristics of Mogamulizumab‐Related AEs

In this study, we retrieved a total of 9,033,057 adverse event reports (AER) from the FAERS database, covering the period from the last quarter of 2018 to the last quarter of 2023. Among these reports, 1182 confirmed mogamulizumab as the primary suspected drug responsible for AEs. Notably, the majority of reports (95.01%) were submitted by healthcare professionals rather than consumers. The United States accounted for the highest proportion of reports (58.71%), followed by Japan (17.85%) and France (8.29%). Regarding clinical outcomes, AEs leading to hospitalization or prolonged hospital stay were the most frequently reported (22.23%), in addition to undisclosed severe AEs. Moreover, a total of 197 reports (19.47%) involved death. Notwithstanding, it is crucial to highlight that a substantial amount of data lacked age and gender information, thus limiting our comprehensive understanding of the relationship between age, gender, and AEs. Refer to Table 1 for further details.

**TABLE 1:** Basic information on adverse events related to mogamulizumab from the FAERS database.

Variable	Total
Year	
2018	19 (1.61%)
2019	209 (17.68%)
2020	193 (16.33%)
2021	254 (21.49%)
2022	326 (27.58%)
2023	181 (15.31%)
Reporter	
Pharmacist	722 (61.08%)
Physician	240 (20.30%)
Other health‐professional	161 (13.62%)
Consumer	58 (4.91%)
Unknown	1 (0.08%)
Reported countries	
United States	694 (58.71%)
Japan	211 (17.85%)
France	98 (8.29%)
Other	179 (15.14%)
Outcomes	
Hospitalization	225 (22.23%)
Death	197 (19.47%)
Life threatening	35 (3.46%)
Disability	9 (0.89%)
Other serious	546 (53.95%)

### 3.2 Signal Detects at the SOC Level

Table 2 presents the signal strengths of mogamulizumab‐related AEs classified by SOC. Based on our statistical analysis, AEs related to mogamulizumab occurred in 22 organ systems. Within these organ systems, several important SOC categories were identified through the screening process, all of which met the criteria of having at least one indicator satisfying the standard among the four analyzed indicators. Significant SOCs included skin and subcutaneous tissue disease (cases = 793, ROR 4.37 [95% CI 4.04–4.73]); infections and invasions (cases = 276, ROR 1.35 [95% CI 1.2–1. 53]); blood and lymphatic system disorders (case = 155, ROR 2.49 [95% CI 2.12–2.93]); metabolism and nutrition disorders (cases = 105, ROR 1.45 [95% CI 1.2–1.77]); immune system disorders (cases = 88, ROR 1.92 [95% CI 1.56–2.38]); and hepatobiliary disorders (cases = 67, ROR 2.17 [95% CI 1.71–2.77]).

**TABLE 2:** The signal strength of adverse events of mogamulizumab at the SOC level in FAERS database.

SOC	Number of cases (*n*)	ROR (95% CI)	PRR (95% CI)	Chi‐squared	IC (IC025)	EBGM (EBGM05)
Skin and subcutaneous tissue disorders	793	4.37 (4.04, 4.73)	3.63 (3.42, 3.85)	1606.29	1.86 (1.75)	3.63 (3.4)
General disorders and administration site conditions	701	1.07 (0.99, 1.17)	1.06 (1, 1.12)	2.93	0.08 (−0.03)	1.06 (0.99)
Infections and infestations	276	1.35 (1.2, 1.53)	1.33 (1.18, 1.5)	23.41	0.41 (0.23)	1.33 (1.2)
Injury, poisoning, and procedural complications	250	0.53 (0.47, 0.61)	0.57 (0.51, 0.64)	94.13	−0.82 (−1)	0.57 (0.51)
Gastrointestinal disorders	226	0.74 (0.65, 0.85)	0.76 (0.68, 0.85)	18.62	−0.4 (−0.59)	0.76 (0.68)
Investigations	218	1 (0.87, 1.14)	1 (0.87, 1.15)	0	0 (−0.2)	1 (0.89)
Blood and lymphatic system disorders	155	2.49 (2.12, 2.93)	2.43 (2.08, 2.84)	132.75	1.28 (1.05)	2.43 (2.12)
Nervous system disorders	139	0.48 (0.4, 0.57)	0.5 (0.43, 0.58)	76.34	−1.01 (−1.25)	0.5 (0.43)
Musculoskeletal and connective tissue disorders	109	0.56 (0.46, 0.67)	0.57 (0.48, 0.68)	37.66	−0.81 (−1.09)	0.57 (0.48)
Metabolism and nutrition disorders	105	1.45 (1.2, 1.77)	1.44 (1.18, 1.75)	14.47	0.53 (0.25)	1.44 (1.23)
Respiratory, thoracic, and mediastinal disorders	103	0.59 (0.49, 0.72)	0.6 (0.49, 0.73)	28.03	−0.73 (−1.01)	0.6 (0.51)
Immune system disorders	88	1.92 (1.56, 2.38)	1.9 (1.53, 2.36)	38.1	0.93 (0.62)	1.9 (1.59)
Cardiac disorders	85	1.13 (0.91, 1.4)	1.13 (0.91, 1.4)	1.28	0.17 (−0.13)	1.13 (0.94)
Psychiatric disorders	73	0.34 (0.27, 0.43)	0.36 (0.28, 0.46)	89.73	−1.49 (−1.82)	0.36 (0.29)
Hepatobiliary disorders	67	2.17 (1.71, 2.77)	2.15 (1.7, 2.72)	41.72	1.11 (0.76)	2.15 (1.76)
Neoplasms benign, malignant, and unspecified (incl cysts and polyps)	53	0.37 (0.28, 0.48)	0.38 (0.29, 0.5)	57.33	−1.41 (−1.8)	0.38 (0.3)
Vascular disorders	52	0.73 (0.56, 0.96)	0.73 (0.55, 0.96)	5.07	−0.44 (−0.84)	0.73 (0.58)
Renal and urinary disorders	52	0.66 (0.5, 0.87)	0.67 (0.51, 0.88)	8.87	−0.59 (−0.98)	0.67 (0.53)
Eye disorders	31	0.43 (0.3, 0.61)	0.43 (0.3, 0.61)	23.65	−1.21 (−1.71)	0.43 (0.32)
Ear and labyrinth disorders	17	1.09 (0.68, 1.76)	1.09 (0.68, 1.74)	0.13	0.13 (−0.54)	1.09 (0.73)
Endocrine disorders	14	1.42 (0.84, 2.41)	1.42 (0.84, 2.41)	1.76	0.51 (−0.22)	1.42 (0.92)
Reproductive system and breast disorders	7	0.29 (0.14, 0.62)	0.3 (0.14, 0.63)	11.86	−1.76 (−2.76)	0.3 (0.16)

### 3.3 Signal of PTs

**FIGURE 2:**
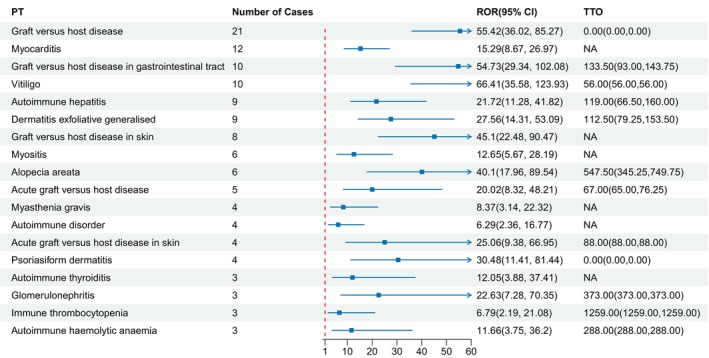
The signal strength of adverse events for mogamulizumab‐associated autoimmune diseases. PT, preferred term; ROR, reporting odds ratio; TTO, time to onset.

A total of 92 AEs related to mogamulizumab have been identified, covering 16 SOCs, all meeting the criteria of four algorithms (Table S3). After excluding AEs potentially caused by the disease itself, which could lead to inaccurate reporting (e.g., disease progression and pruritus), the top five PTs in terms of number of cases were rash (*n* = 218), infusion‐related reaction (*n* = 95), pyrexia (*n* = 73), erythema (*n* = 52), and drug eruption (*n* = 48). Table 3 displays the top 40 PTs sorted by ROR. Upon ranking by descending ROR values, the top five PTs are tumor flare (ROR 256.55 [95% CI 126.42–520.63]), cytomegalovirus enteritis (ROR = 244.37 [95% CI 114.76–520.32]), bulbar palsy (ROR = 238.36 [95% CI 75.22–755.31]), cytomegalovirus enterocolitis (ROR = 124.39 [95% CI 55.41–279.27]), and vitiligo (ROR = 66.41[95% CI 35.58–123.93]). Notably, several unexpectedly significant AEs were also identified, including pemphigoid (ROR = 5.69[95% CI 1.83–17.66]), unstable angina (ROR = 20.56 [95% CI 8.54–49.5]), bulbar palsy (ROR = 238.36 [95% CI 75.22–755.31]), myositis (ROR = 12.65 [95% CI 5.67–28.19]), and several autoimmune diseases, including autoimmune hepatitis (ROR = 21.33 [95% CI 11.08–41.07]), myocarditis (ROR = 15.29 [95% CI 8.67–26.97]), glomerulonephritis (ROR = 22.49 [95% CI 7.24–69.9]), nephrotic syndrome (ROR = 7.63 [95% CI 2.46–23.67]), myasthenia gravis (ROR = 8.54 [95% CI 3.2–22.77]), and autoimmune thyroiditis (ROR = 11.81 [95% CI 3.8–36.68]), as shown in Figure 2. Additionally, mogamulizumab was found to be associated with leukopenia (ROR = 3.35 [95% CI 2.24–5.01]), neutropenia (ROR = 8.4 [95% CI 6.02–11.72]), and thrombocytopenia (ROR = 6.79 [95% CI 2.19–21.08]).

**TABLE 3:** The top 40 signal strength of adverse events of mogamulizumab ranked by ROR at the PT level in FAERS database.

PT	Number of cases (*n*)	ROR (95% CI)	PRR (95% CI)	Chi‐squared	IC (IC025)	EBGM (EBGM05)
Tumor flare	8	256.55 (126.42, 520.63)	255.98 (126.41, 518.38)	1952.23	7.94 (6.98)	245.98 (136.06)
Cytomegalovirus enteritis	7	244.37 (114.76, 520.32)	243.89 (115.81, 513.64)	1629.92	7.88 (6.85)	234.8 (124.76)
Bulbar palsy	3	238.36 (75.22, 755.31)	238.16 (74.93, 756.98)	682.58	7.84 (6.39)	229.48 (87.43)
Cytomegalovirus enterocolitis	6	124.39 (55.41, 279.27)	124.19 (55.6, 277.39)	718.96	6.93 (5.85)	121.8 (61.91)
Vitiligo	10	66.41 (35.58, 123.93)	66.23 (35.37, 124.01)	635.72	6.03 (5.18)	65.54 (38.89)
Graft‐versus‐host disease	21	55.42 (36.02, 85.27)	55.11 (35.81, 84.82)	1106.01	5.77 (5.16)	54.63 (38.1)
Graft‐versus‐host disease in gastrointestinal tract	10	54.73 (29.34, 102.08)	54.58 (29.15, 102.19)	521.5	5.76 (4.9)	54.12 (32.12)
Drug eruption	48	48.45 (36.4, 64.48)	47.82 (36.34, 62.92)	2184.39	5.57 (5.16)	47.47 (37.37)
Graft‐versus‐host disease in skin	8	45.1 (22.48, 90.47)	45 (22.66, 89.36)	341.73	5.48 (4.53)	44.68 (24.96)
Cytomegalovirus test positive	4	40.31 (15.07, 107.8)	40.27 (15.11, 107.3)	152.19	5.32 (4.05)	40.02 (17.57)
Alopecia areata	6	40.1 (17.96, 89.54)	40.03 (17.92, 89.41)	226.9	5.31 (4.24)	39.78 (20.31)
Granuloma	6	32.89 (14.73, 73.41)	32.84 (14.7, 73.35)	184.24	5.03 (3.96)	32.67 (16.69)
Psoriasiform dermatitis	4	30.48 (11.41, 81.44)	30.44 (11.42, 81.11)	113.36	4.92 (3.65)	30.3 (13.31)
Infusion site reaction	4	28.77 (10.77, 76.86)	28.74 (10.79, 76.58)	106.6	4.84 (3.57)	28.61 (12.57)
Cytomegalovirus chorioretinitis	4	28.7 (10.74, 76.68)	28.67 (10.76, 76.39)	106.34	4.84 (3.57)	28.54 (12.54)
Generalized exfoliative dermatitis	9	27.56 (14.31, 53.09)	27.49 (14.4, 52.49)	228.79	4.77 (3.88)	27.38 (15.82)
Acute graft‐versus‐host disease in skin	4	25.06 (9.38, 66.95)	25.04 (9.4, 66.72)	91.94	4.64 (3.37)	24.94 (10.96)
Angina unstable	5	24.7 (10.26, 59.49)	24.67 (10.21, 59.6)	113.12	4.62 (3.46)	24.58 (11.78)
Glomerulonephritis	3	22.63 (7.28, 70.35)	22.61 (7.25, 70.47)	61.75	4.49 (3.08)	22.54 (8.72)
Autoimmune hepatitis	9	21.72 (11.28, 41.82)	21.67 (11.35, 41.38)	176.83	4.43 (3.54)	21.6 (12.48)
Cytomegalovirus viremia	7	21.26 (10.11, 44.68)	21.22 (10.08, 44.69)	134.42	4.4 (3.4)	21.15 (11.36)
Infusion‐related reaction	95	20.96 (17.09, 25.71)	20.43 (16.79, 24.85)	1752.52	4.35 (4.06)	20.37 (17.17)
Acute graft‐versus‐host disease	5	20.02 (8.32, 48.21)	20 (8.28, 48.31)	89.96	4.32 (3.16)	19.94 (9.56)
Hypoalbuminemia	8	19.49 (9.73, 39.04)	19.45 (9.79, 38.62)	139.57	4.28 (3.33)	19.39 (10.84)
Skin erosion	4	18.49 (6.93, 49.37)	18.47 (6.93, 49.21)	65.92	4.2 (2.94)	18.42 (8.1)
Lymphopenia	18	18.24 (11.47, 29)	18.15 (11.34, 29.05)	290.97	4.18 (3.53)	18.1 (12.28)
Hyperuricemia	4	17.57 (6.58, 46.91)	17.56 (6.59, 46.79)	62.28	4.13 (2.86)	17.51 (7.7)
Cytomegalovirus infection reactivation	6	16.63 (7.46, 37.09)	16.61 (7.44, 37.1)	87.77	4.05 (2.98)	16.56 (8.47)
Skin disorder	35	16.53 (11.84, 23.07)	16.38 (11.74, 22.86)	504.41	4.03 (3.56)	16.34 (12.36)
Skin lesion	28	16.32 (11.24, 23.68)	16.2 (11.16, 23.51)	398.44	4.01 (3.49)	16.16 (11.83)
Myocarditis	12	15.29 (8.67, 26.97)	15.24 (8.63, 26.91)	159.36	3.93 (3.14)	15.21 (9.46)
Cytomegalovirus infection	16	14.59 (8.92, 23.86)	14.53 (8.9, 23.72)	201.21	3.86 (3.17)	14.5 (9.61)
Skin plaque	10	14.29 (7.68, 26.6)	14.25 (7.61, 26.68)	122.95	3.83 (2.97)	14.22 (8.45)
Erythema multiforme	6	13.9 (6.23, 30.98)	13.88 (6.21, 31)	71.53	3.79 (2.72)	13.85 (7.08)
Skin weeping	3	13.23 (4.26, 41.1)	13.22 (4.24, 41.2)	33.82	3.72 (2.31)	13.2 (5.11)
Myositis	6	12.65 (5.67, 28.19)	12.63 (5.65, 28.21)	64.12	3.66 (2.58)	12.6 (6.44)
Hypercalcemia	9	12.46 (6.48, 23.99)	12.44 (6.52, 23.75)	94.47	3.63 (2.74)	12.41 (7.18)
Autoimmune thyroiditis	3	12.05 (3.88, 37.41)	12.04 (3.86, 37.53)	30.31	3.59 (2.17)	12.02 (4.66)
Autoimmune hemolytic anemia	3	11.66 (3.75, 36.2)	11.65 (3.74, 36.31)	29.16	3.54 (2.12)	11.63 (4.51)
Stevens–Johnson syndrome	10	11.22 (6.03, 20.89)	11.19 (5.98, 20.95)	92.68	3.48 (2.63)	11.18 (6.65)

### 3.4 Onset Time of Mogamulizumab‐Related AEs

We obtained data on the occurrence time of AEs related to mogamulizumab from the FAERS database. After excluding erroneous and missing reports, a total of 255 reports provided accurate information on the onset time. According to the data, the median onset time for AEs was 21 days, with an interquartile range (IQR) of 2–107 days. As shown in Figure S1, 33.73% of patients experienced adverse reactions within the first week of using mogamulizumab, and 20.78% of patients experienced adverse reactions within the first month. The probability of experiencing AEs was lowest in the second month of treatment, at only 9.80%. However, after 2 months, the probability of AEs significantly increased to 35.69%. These findings suggest the necessity of monitoring potential adverse reactions in patients even after several months of mogamulizumab treatment.

## 4 Discussion

It is worth pointing out that previous safety studies of mogamulizumab have been limited to clinical trials or have focused solely on specific AEs such as rash. This narrow focus may not provide a comprehensive understanding of potential safety concerns. In this large‐scale study of real‐world drug surveillance, we have identified previously unknown AEs associated with mogamulizumab. These newly identified AEs encompass pemphigoid, myocarditis, bulbar palsy, myositis, myasthenia gravis, among others, in addition to the AEs already documented on the drug label. To our knowledge, this is the first pharmacovigilance analysis conducted after the market approval of mogamulizumab, providing valuable insights for further optimizing its clinical usage.

At the organ level, our study found that the most common AEs associated with mogamulizumab occurred in the “skin and subcutaneous tissue disorders” category. Previous clinical trials mainly reported skin‐related AEs such as rash [16], with few case reports of other conditions including vitiligo [17], alopecia areata [18], psoriasiform dermatitis [19], toxic epidermal necrolysis [20], and photosensitivity reaction [21]. These AEs were also confirmed in our study. Additionally, our research suggested some previously unreported skin manifestations, such as pemphigus and bullae formation. Mechanistically, while the mechanism of action of mogamulizumab targeting CCR4 can effectively reduce the malignant T‐cell burden and promote antitumor immunity by depleting Treg cells in the tumor microenvironment, it also leads to hyperactivation of the Th1 immune response. Some researchers have found a reduction in circulating and skin‐infiltrating Tregs during mogamulizumab treatment, leading to severe inflammatory and immune‐mediated skin disorders [22]. Although some studies suggest that certain types of skin lesions might be markers for better tumor prognosis [23], a larger‐scale observational study is needed to assess the impact of mogamulizumab‐induced skin AEs, such as alopecia areata, on tumor prognosis [19]. Moreover, previous studies indicated that 7% of patients receiving mogamulizumab therapy discontinued treatment due to drug eruption [7], and severe skin conditions causing physical appearance changes could result in significant psychological trauma for patients. This emphasizes the need for further research to identify reliable biomarkers for predicting and managing mogamulizumab‐induced skin manifestations.

Similar to many monoclonal antibody drugs, mogamulizumab was found to be associated with infectious events, including cytomegalovirus infection, staphylococcal infection, and fungal infection (candidiasis). However, it is crucial to note that in T‐cell lymphoma patients who have not received mogamulizumab treatment, the incidence of staphylococcal infections and sepsis is also present. Furthermore, due to limitations in the FAERS database, there may be reporting errors in AEs related to bacterial infections as identified in this study. CCR4 is expressed in Th17 cells, Tregs, and T‐helper type 2 cells, which play important roles in maintaining immune homeostasis [1]. As a monoclonal antibody targeting CCR4, mogamulizumab partially impairs the functions of these immune cells, thereby affecting normal anti‐infection responses in the body.

Furthermore, we also observed the impact of mogamulizumab on the hematological system. Mogamulizumab was found to be associated with leukopenia, neutropenia, and thrombocytopenia and could potentially cause severe coagulation abnormalities such as disseminated intravascular coagulation. It may also result in hypercalcemia, hyperuricemia, and other metabolic disturbances associated with tumor destruction, tumor metabolic disruption, and enhanced immune response. Close monitoring of changes in blood biochemical indicators is vital for timely management during clinical practice.

We also observed new signals related to self‐immune enhancement, including autoimmune hepatitis, myocarditis, glomerulonephritis, nephrotic syndrome, myasthenia gravis, and autoimmune thyroiditis. The specific mechanisms underlying these side effects remain to be elucidated, and predicting the occurrence of these AEs is a challenging goal.

Although this study provides reliable scientific evidence for the safety evaluation of mogamulizumab, all pharmacovigilance databases have inherent flaws [24, 25]. Firstly, disproportionality analysis can only assess the strength of signals and explore potential associations, but cannot determine causal relationships. Secondly, due to the phenomena of underreporting and overreporting, accurately quantifying the real risk in clinical practice is challenging, and the incidence rates calculated based on spontaneous reports are not precise. Additionally, the data analysis did not consider various unmeasured confounding factors that may affect AEs, such as potential drug interactions, adjustments in treatment regimens, and laboratory and instrument testing. Therefore, further experimental and prospective clinical studies are necessary to validate these findings.

## 5 Conclusion

Using real‐world data from the FAERS database, we performed pharmacovigilance analysis to identify safety signals and potential risks related to the utilization of mogamulizumab. The AEs observed in this study exhibited a general consistency with those indicated in the prescribing information; however, several unexpected and significant AEs were also detected, including autoimmune diseases like autoimmune hepatitis, myocarditis, and glomerulonephritis, as well as disseminated intravascular coagulation. These results, characterized by robust signal‐like AEs, serve to partially offset the limitations imposed by the small sample size in the clinical studies of this drug. However, given the presence of population heterogeneity, incomplete data, reporting bias, and other potential factors that may impact the analysis results, additional basic research and prospective clinical studies are still required to validate and interpret the relationship between reporting bias and these AEs. The findings of this study contribute to the investigation of rare AEs, augment the FAERS database system, and offer an innovative perspective for the identification of analogous events in the future.

## Author Contributions

**Genshan Zhang:** data curation (equal), formal analysis (equal), methodology (equal), project administration (equal), validation (equal), visualization (equal), writing – original draft (lead), writing – review and editing (equal). **Haokun Zhang:** formal analysis (equal), writing – review and editing (equal). **Jie Fu:** data curation (equal), supervision (equal), validation (equal), visualization (equal), writing – review and editing (equal). **Zhixin Cao:** funding acquisition (lead), project administration (equal), writing – review and editing (equal).

## Conflicts of Interest

The authors declare no conflicts of interest.

## Supporting information


Data S1.

## Data Availability

The original data involved in this analysis can be downloaded from http://www.fda.gov/Safety/MedWatch/, while other data can be obtained by contacting the corresponding author.

## References

[1] O. Yoshie and K. Matsushima , “CCR4 and Its Ligands: From Bench to Bedside,” International Immunology 27 (2015): 11–20. 25087232 10.1093/intimm/dxu079

[2] O. Yoshie , “CCR4 as a Therapeutic Target for Cancer Immunotherapy,” Cancers 13 (2021): 5542. 34771703 10.3390/cancers13215542 PMC8583476

[3] J. P. Nicolay , J. D. Albrecht , S. Alberti‐Violetti , and E. Berti , “CCR4 in Cutaneous T‐Cell Lymphoma: Therapeutic Targeting of a Pathogenic Driver,” European Journal of Immunology 51 (2021): 1660–1671. 33811642 10.1002/eji.202049043

[4] M. Roelens , A. de Masson , A. Andrillon , et al., “Mogamulizumab Induces Long‐Term Immune Restoration and Reshapes Tumour Heterogeneity in Sézary Syndrome,” British Journal of Dermatology 186 (2022): 1010–1025. 35041763 10.1111/bjd.21018

[5] Y. H. Kim , M. Bagot , L. Pinter‐Brown , et al., “Mogamulizumab Versus Vorinostat in Previously Treated Cutaneous T‐Cell Lymphoma (MAVORIC): An International, Open‐Label, Randomised, Controlled Phase 3 Trial,” Lancet Oncology 19 (2018): 1192–1204. 30100375 10.1016/S1470-2045(18)30379-6

[6] M. Fernández‐Guarino , P. Ortiz , F. Gallardo , and M. Llamas‐Velasco , “Clinical and Real‐World Effectiveness of Mogamulizumab: A Narrative Review,” International Journal of Molecular Sciences 25 (2024): 2203. 38396877 10.3390/ijms25042203 PMC10889597

[7] Y. L. Kasamon , H. Chen , R. A. de Claro , et al., “FDA Approval Summary: Mogamulizumab‐Kpkc for Mycosis Fungoides and Sézary Syndrome,” Clinical Cancer Research 25 (2019): 7275–7280. 31366601 10.1158/1078-0432.CCR-19-2030

[8] D. C. Moore , J. B. Elmes , P. A. Shibu , C. Larck , and S. I. Park , “Mogamulizumab: An Anti‐CC Chemokine Receptor 4 Antibody for T‐Cell Lymphomas,” Annals of Pharmacotherapy 54 (2020): 371–379. 31648540 10.1177/1060028019884863

[9] G. S. Silva , E. J. Kim , S. K. Barta , and J. Chung , “Immune‐Related Adverse Events Associated With Mogamulizumab: A Comprehensive Review of the Literature,” Expert Review of Anticancer Therapy 24 (2024): 819–827. 38990648 10.1080/14737140.2024.2379914

[10] T. Sugio , K. Kato , T. Aoki , et al., “Mogamulizumab Treatment Prior to Allogeneic Hematopoietic Stem Cell Transplantation Induces Severe Acute Graft‐Versus‐Host Disease,” Biology of Blood and Marrow Transplantation 22 (2016): 1608–1614. 27220263 10.1016/j.bbmt.2016.05.017

[11] FDA Adverse Event Reporting System (FAERS) , “FDA Adverse Event Reporting System (FAERS) Database,” 2021, https://www.fda.gov/drugs/drug‐approvals‐and‐databases/fda‐adverse‐event‐reporting‐system‐faers.

[12] E. G. Brown , “Using MedDRA: Implications for Risk Management,” Drug Safety 27 (2004): 591–602. 15154830 10.2165/00002018-200427080-00010

[13] K. J. Rothman , S. Lanes , and S. T. Sacks , “The Reporting Odds Ratio and Its Advantages Over the Proportional Reporting Ratio,” Pharmacoepidemiology and Drug Safety 13 (2004): 519–523. 15317031 10.1002/pds.1001

[14] S. J. Evans , P. C. Waller , and S. Davis , “Use of Proportional Reporting Ratios (PRRs) for Signal Generation From Spontaneous Adverse Drug Reaction Reports,” Pharmacoepidemiology and Drug Safety 10 (2001): 483–486. 11828828 10.1002/pds.677

[15] W. Dumouchel , “Bayesian Data Mining in Large Frequency Tables, With an Application to the FDA Spontaneous Reporting System,” American Statistician 53 (1999): 177–190.

[16] A. C. M. Musiek , K. E. Rieger , M. Bagot , et al., “Dermatologic Events Associated With the Anti‐CCR4 Antibody Mogamulizumab: Characterization and Management,” Dermatology and Therapy 12 (2022): 29–40. 34816383 10.1007/s13555-021-00624-7 PMC8776934

[17] A. S. Algarni , C. Ram‐Wolff , M. Bagot , and A. De Masson , “Mogamulizumab‐Induced Vitiligo in Patients With Sézary Syndrome: Three Cases,” European Journal of Dermatology 31 (2021): 213–216. 33814357 10.1684/ejd.2021.4002

[18] N. S. Raval , N. A. Alexander , K. De Monnin , et al., “Alopecia Areata After Mogamulizumab Treatment,” JAAD Case Reports 19 (2022): 68–70. 34917728 10.1016/j.jdcr.2021.10.034 PMC8669261

[19] G. Avallone , G. Roccuzzo , A. Pileri , et al., “Clinicopathological Definition, Management and Prognostic Value of Mogamulizumab‐Associated Rash and Other Cutaneous Events: A Systematic Review,” Journal of the European Academy of Dermatology and Venereology 38 (2024): 1738–1748, 10.1111/jdv.19801. 38279614

[20] I. Lazaridou , J. Calvani , E. Annabi , et al., “Toxic Epidermal Necrolysis Possibly Associated With Mogamulizumab in a Patient With Sézary Syndrome,” Journal of the European Academy of Dermatology and Venereology 37 (2023): e715–e717. 36645855 10.1111/jdv.18868

[21] Y. Masuda , K. Tatsuno , S. Kitano , et al., “Mogamulizumab‐Induced Photosensitivity in Patients With Mycosis Fungoides and Other T‐Cell Neoplasms,” Journal of the European Academy of Dermatology and Venereology 32 (2018): 1456–1460. 29341283 10.1111/jdv.14797

[22] A. Rajendiran and K. Tenbrock , “Regulatory T Cell Function in Autoimmune Disease,” Journal of Translational Autoimmunity 4 (2021): 100130. 35005594 10.1016/j.jtauto.2021.100130 PMC8716637

[23] K. Yonekura , T. Kanzaki , K. Gunshin , et al., “Effect of Anti‐CCR4 Monoclonal Antibody (Mogamulizumab) on Adult T‐Cell Leukemia‐Lymphoma: Cutaneous Adverse Reactions May Predict the Prognosis,” Journal of Dermatology 41 (2014): 239–244. 24628073 10.1111/1346-8138.12419

[24] P. Ma , H. Tian , Q. Shi , et al., “High Risks Adverse Events Associated With Trastuzumab Emtansine and Trastuzumab Deruxtecan for the Treatment of HER2‐Positive/Mutated Malignancies: A Pharmacovigilance Study Based on the FAERS Database,” Expert Opinion on Drug Safety 22 (2023): 685–696. 37068935 10.1080/14740338.2023.2204228

[25] Z. Feng , Q. Zhao , J. Wu , et al., “Nonselective Beta‐Adrenoceptor Blocker Use and Risk of Parkinson's Disease: From Multiple Real‐World Evidence,” BMC Medicine 21 (2023): 437. 37964359 10.1186/s12916-023-03122-z PMC10647086

