# Supplementary material for: Mogamulizumab‐Associated Autoimmune Diseases: Insights From FAERS Database Analysis
Source: Cancer Med. 2024 Dec 10;13(23):e70478. doi: 10.1002/cam4.70478 (PMC11632115; doi:10.1002/cam4.70478)
Supplement: Supplementary file 1 — Data S1. [file CAM4-13-e70478-s001.docx]

**Supplementary Materials**

[Figure S1. Time to onset of mogamulizumab-associated AEs. 2](#_Toc179031091)

[Table S1. Two-by-two contingency table for disproportionality analyses 3](#_Toc179031092)

[Table S2. The specific formulas for the four algorithms 4](#_Toc179031093)

[Table S3. Signal strength of adverse events of Mogamulizumab ranked by ROR at the PTs level in FAERS database 5](#_Toc179031094)

Figure S1. Time to onset of mogamulizumab-associated AEs.

(A) Proportions of cases based on the time to onset of mogamulizumab-associated AEs. (B) Number of cases based on the time to onset of mogamulizumab-associated AEs.


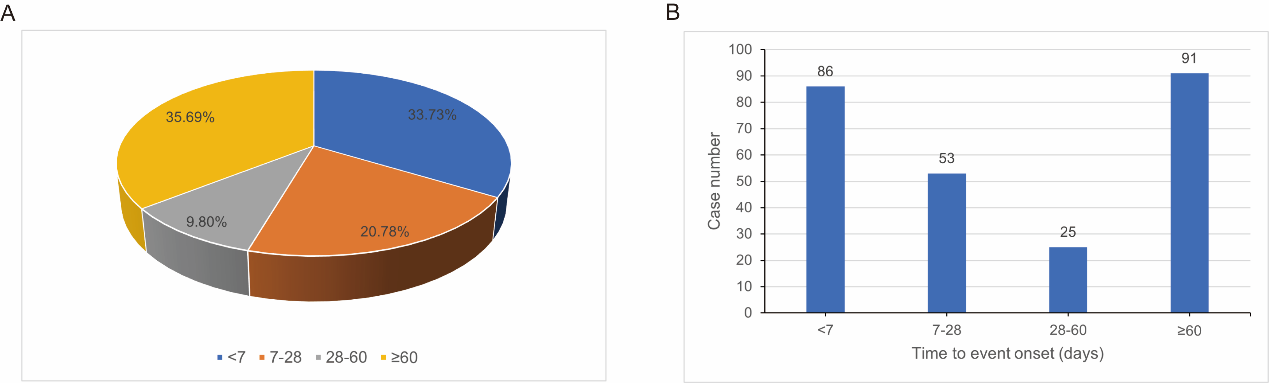


Table S1. Two-by-two contingency table for disproportionality analyses

| **Type of drug** | **N of target adverse reaction reports** | **N of other adverse reaction reports** | **Total** |
| --- | --- | --- | --- |
| Target drug | a | b | a+b |
| Other drugs | c | d | c+d |
| Total | a+c | b+d | a+b+c+d |

Table S2. The specific formulas for the four algorithms

| **Algorithms** | **Equation** | **Criteria** |
| --- | --- | --- |
| ROR | ROR = ad/bc | Lower limit of 95% CI >1, N≥3 |
|  | 95%CI = e^ln(ROR)±1.96(1/a+1/b/+1/c+1/d)^0.5^ |  |
| PRR | PRR = [a(c+d)]/[c(a+b)] | PRR≥2, χ^2^≥4, N≥3 |
|  | χ^2^= [(ad-bc)^2](a+b+c+d)/[(a+b)(c+d)(a+c)(b+d)] |  |
| BCPNN | IC = $\text{log}_{\text{2}} \text{a(a+b+c+d)/[(a+c)(a+b)]}$ | IC025>0 |
|  | 95%CI = E(IC) ± 2[V(IC)]^0.5 |  |
| MGPS | EBGM = a(a+b+c+d)/[(a+c)(a+b)] | EBGM05>2 |
|  | 95%CI = $\text{e}^{\text{ln(EBGM)±1.96(1/a+1/b/+1/c+1/d)\textasciicircum0.5}}$ |  |

**Notes:** Equation: a, number of reports containing both the target drug and the target adverse drug reaction; b, number of reports containing other adverse drug reactions of the target drug; c, number of reports containing the target adverse drug reaction of other drugs; d, number of reports containing other drugs and other adverse drug reactions.

The MGPS employs an empirical Bayesian approach, whereby a prior distribution is obtained by maximum likelihood estimates, and the prior and likelihood are subsequently combined to obtain a posterior distribution. The fifth percentile of the posterior distribution is denoted by “EBGM05” and is interpreted as the one-sided 95% confidence lower bound for the EBGM.

**Abbreviations:** ROR, reporting odds ratio; 95% CI, 95% confidence interval; N, the number of reports; PRR, proportional reporting ratio; χ2, chi-squared; BCPNN, bayesian confidence propagation neural network; IC, information component; IC025, the lower 95% CI of the IC; E (IC), the IC expectations; V (IC), the variance of IC; MGPS, multi-item gamma Poisson shrinker; EBGM, empirical Bayesian geometric mean; EBGM05, the lower 95% CI of EBGM.

Table S3. Signal strength of adverse events of Mogamulizumab ranked by ROR at the PTs level in FAERS database

| **PT** | **Number of Cases (n)** | **ROR(95% CI)** | **PRR(95% CI)** | **chisq** | **IC(IC025)** | **EBGM(EBGM05)** |
| --- | --- | --- | --- | --- | --- | --- |
| tumour flare | 8 | 256.55(126.42, 520.63) | 255.98(126.41, 518.38) | 1952.23 | 7.94(6.98) | 245.98(136.06) |
| cytomegalovirus enteritis | 7 | 244.37(114.76, 520.32) | 243.89(115.81, 513.64) | 1629.92 | 7.88(6.85) | 234.8(124.76) |
| bulbar palsy | 3 | 238.36(75.22, 755.31) | 238.16(74.93, 756.98) | 682.58 | 7.84(6.39) | 229.48(87.43) |
| tumour invasion | 4 | 159.96(59.26, 431.76) | 159.79(58.81, 434.18) | 615.49 | 7.28(6) | 155.84(67.9) |
| cytomegalovirus enterocolitis | 6 | 124.39(55.41, 279.27) | 124.19(55.6, 277.39) | 718.96 | 6.93(5.85) | 121.8(61.91) |
| vitiligo | 10 | 66.41(35.58, 123.93) | 66.23(35.37, 124.01) | 635.72 | 6.03(5.18) | 65.54(38.89) |
| graft versus host disease | 21 | 55.42(36.02, 85.27) | 55.11(35.81, 84.82) | 1106.01 | 5.77(5.16) | 54.63(38.1) |
| graft versus host disease in gastrointestinal tract | 10 | 54.73(29.34, 102.08) | 54.58(29.15, 102.19) | 521.5 | 5.76(4.9) | 54.12(32.12) |
| drug eruption | 48 | 48.45(36.4, 64.48) | 47.82(36.34, 62.92) | 2184.39 | 5.57(5.16) | 47.47(37.37) |
| staphylococcal skin infection | 3 | 46.96(15.07, 146.28) | 46.92(15.05, 146.24) | 133.82 | 5.54(4.12) | 46.58(18) |
| graft versus host disease in skin | 8 | 45.1(22.48, 90.47) | 45(22.66, 89.36) | 341.73 | 5.48(4.53) | 44.68(24.96) |
| cytomegalovirus test positive | 4 | 40.31(15.07, 107.8) | 40.27(15.11, 107.3) | 152.19 | 5.32(4.05) | 40.02(17.57) |
| alopecia areata | 6 | 40.1(17.96, 89.54) | 40.03(17.92, 89.41) | 226.9 | 5.31(4.24) | 39.78(20.31) |
| granuloma | 6 | 32.89(14.73, 73.41) | 32.84(14.7, 73.35) | 184.24 | 5.03(3.96) | 32.67(16.69) |
| dermatitis psoriasiform | 4 | 30.48(11.41, 81.44) | 30.44(11.42, 81.11) | 113.36 | 4.92(3.65) | 30.3(13.31) |
| infusion site reaction | 4 | 28.77(10.77, 76.86) | 28.74(10.79, 76.58) | 106.6 | 4.84(3.57) | 28.61(12.57) |
| cytomegalovirus chorioretinitis | 4 | 28.7(10.74, 76.68) | 28.67(10.76, 76.39) | 106.34 | 4.84(3.57) | 28.54(12.54) |
| dermatitis exfoliative generalised | 9 | 27.56(14.31, 53.09) | 27.49(14.4, 52.49) | 228.79 | 4.77(3.88) | 27.38(15.82) |
| acute graft versus host disease in skin | 4 | 25.06(9.38, 66.95) | 25.04(9.4, 66.72) | 91.94 | 4.64(3.37) | 24.94(10.96) |
| angina unstable | 5 | 24.7(10.26, 59.49) | 24.67(10.21, 59.6) | 113.12 | 4.62(3.46) | 24.58(11.78) |
| glomerulonephritis | 3 | 22.63(7.28, 70.35) | 22.61(7.25, 70.47) | 61.75 | 4.49(3.08) | 22.54(8.72) |
| autoimmune hepatitis | 9 | 21.72(11.28, 41.82) | 21.67(11.35, 41.38) | 176.83 | 4.43(3.54) | 21.6(12.48) |
| cytomegalovirus viraemia | 7 | 21.26(10.11, 44.68) | 21.22(10.08, 44.69) | 134.42 | 4.4(3.4) | 21.15(11.36) |
| infusion related reaction | 95 | 20.96(17.09, 25.71) | 20.43(16.79, 24.85) | 1752.52 | 4.35(4.06) | 20.37(17.17) |
| acute graft versus host disease | 5 | 20.02(8.32, 48.21) | 20(8.28, 48.31) | 89.96 | 4.32(3.16) | 19.94(9.56) |
| hypoalbuminaemia | 8 | 19.49(9.73, 39.04) | 19.45(9.79, 38.62) | 139.57 | 4.28(3.33) | 19.39(10.84) |
| skin erosion | 4 | 18.49(6.93, 49.37) | 18.47(6.93, 49.21) | 65.92 | 4.2(2.94) | 18.42(8.1) |
| lymphopenia | 18 | 18.24(11.47, 29) | 18.15(11.34, 29.05) | 290.97 | 4.18(3.53) | 18.1(12.28) |
| disease progression | 126 | 17.97(15.04, 21.47) | 17.38(14.57, 20.73) | 1943.14 | 4.12(3.86) | 17.33(14.93) |
| hyperuricaemia | 4 | 17.57(6.58, 46.91) | 17.56(6.59, 46.79) | 62.28 | 4.13(2.86) | 17.51(7.7) |
| cytomegalovirus infection reactivation | 6 | 16.63(7.46, 37.09) | 16.61(7.44, 37.1) | 87.77 | 4.05(2.98) | 16.56(8.47) |
| skin disorder | 35 | 16.53(11.84, 23.07) | 16.38(11.74, 22.86) | 504.41 | 4.03(3.56) | 16.34(12.36) |
| skin lesion | 28 | 16.32(11.24, 23.68) | 16.2(11.16, 23.51) | 398.44 | 4.01(3.49) | 16.16(11.83) |
| myocarditis | 12 | 15.29(8.67, 26.97) | 15.24(8.63, 26.91) | 159.36 | 3.93(3.14) | 15.21(9.46) |
| cytomegalovirus infection | 16 | 14.59(8.92, 23.86) | 14.53(8.9, 23.72) | 201.21 | 3.86(3.17) | 14.5(9.61) |
| skin plaque | 10 | 14.29(7.68, 26.6) | 14.25(7.61, 26.68) | 122.95 | 3.83(2.97) | 14.22(8.45) |
| oesophageal candidiasis | 3 | 14.01(4.51, 43.51) | 14(4.49, 43.63) | 36.13 | 3.8(2.39) | 13.97(5.41) |
| erythema multiforme | 6 | 13.9(6.23, 30.98) | 13.88(6.21, 31) | 71.53 | 3.79(2.72) | 13.85(7.08) |
| skin weeping | 3 | 13.23(4.26, 41.1) | 13.22(4.24, 41.2) | 33.82 | 3.72(2.31) | 13.2(5.11) |
| therapy partial responder | 15 | 12.65(7.62, 21.02) | 12.61(7.58, 20.99) | 160 | 3.65(2.94) | 12.58(8.23) |
| myositis | 6 | 12.65(5.67, 28.19) | 12.63(5.65, 28.21) | 64.12 | 3.66(2.58) | 12.6(6.44) |
| hypercalcaemia | 9 | 12.46(6.48, 23.99) | 12.44(6.52, 23.75) | 94.47 | 3.63(2.74) | 12.41(7.18) |
| autoimmune thyroiditis | 3 | 12.05(3.88, 37.41) | 12.04(3.86, 37.53) | 30.31 | 3.59(2.17) | 12.02(4.66) |
| autoimmune haemolytic anaemia | 3 | 11.66(3.75, 36.2) | 11.65(3.74, 36.31) | 29.16 | 3.54(2.12) | 11.63(4.51) |
| stevens-johnson syndrome | 10 | 11.22(6.03, 20.89) | 11.19(5.98, 20.95) | 92.68 | 3.48(2.63) | 11.18(6.65) |
| stress cardiomyopathy | 4 | 11.21(4.2, 29.91) | 11.2(4.2, 29.84) | 37.09 | 3.48(2.22) | 11.18(4.92) |
| toxic skin eruption | 6 | 10.8(4.85, 24.08) | 10.79(4.83, 24.1) | 53.2 | 3.43(2.36) | 10.77(5.51) |
| rash erythematous | 23 | 9.33(6.19, 14.06) | 9.28(6.15, 14.01) | 169.74 | 3.21(2.63) | 9.27(6.57) |
| atrioventricular block complete | 3 | 9.28(2.99, 28.83) | 9.28(2.98, 28.92) | 22.13 | 3.21(1.8) | 9.27(3.59) |
| troponin increased | 4 | 8.94(3.35, 23.86) | 8.93(3.35, 23.79) | 28.15 | 3.16(1.89) | 8.92(3.93) |
| hepatic cytolysis | 8 | 8.88(4.43, 17.77) | 8.86(4.46, 17.59) | 55.72 | 3.15(2.2) | 8.85(4.95) |
| cardiomyopathy | 6 | 8.74(3.92, 19.47) | 8.72(3.9, 19.48) | 40.97 | 3.12(2.05) | 8.71(4.46) |
| blood lactate dehydrogenase increased | 6 | 8.59(3.86, 19.15) | 8.58(3.84, 19.16) | 40.13 | 3.1(2.03) | 8.57(4.38) |
| rash | 218 | 8.53(7.44, 9.78) | 8.08(7.04, 9.27) | 1359.97 | 3.01(2.82) | 8.07(7.19) |
| febrile neutropenia | 35 | 8.4(6.02, 11.72) | 8.33(5.97, 11.62) | 225.7 | 3.06(2.58) | 8.32(6.3) |
| myasthenia gravis | 4 | 8.37(3.14, 22.32) | 8.36(3.14, 22.27) | 25.88 | 3.06(1.8) | 8.35(3.67) |
| tumour lysis syndrome | 5 | 8.33(3.46, 20.05) | 8.32(3.44, 20.1) | 32.19 | 3.06(1.9) | 8.31(3.99) |
| nephrotic syndrome | 3 | 7.9(2.55, 24.53) | 7.9(2.53, 24.62) | 18.05 | 2.98(1.56) | 7.89(3.06) |
| myelosuppression | 18 | 7.84(4.93, 12.46) | 7.81(4.88, 12.5) | 106.77 | 2.96(2.31) | 7.8(5.29) |
| toxic epidermal necrolysis | 6 | 7.41(3.33, 16.52) | 7.4(3.31, 16.53) | 33.2 | 2.89(1.82) | 7.4(3.78) |
| skin mass | 3 | 6.81(2.19, 21.13) | 6.8(2.18, 21.19) | 14.83 | 2.76(1.35) | 6.8(2.63) |
| immune thrombocytopenia | 3 | 6.79(2.19, 21.08) | 6.79(2.18, 21.16) | 14.79 | 2.76(1.35) | 6.78(2.63) |
| disseminated intravascular coagulation | 4 | 6.74(2.53, 17.99) | 6.74(2.53, 17.96) | 19.53 | 2.75(1.48) | 6.73(2.96) |
| hypophosphataemia | 3 | 6.62(2.13, 20.53) | 6.61(2.12, 20.6) | 14.27 | 2.72(1.31) | 6.61(2.56) |
| lymphadenopathy | 13 | 6.51(3.78, 11.23) | 6.49(3.75, 11.24) | 60.4 | 2.7(1.94) | 6.49(4.11) |
| autoimmune disorder | 4 | 6.29(2.36, 16.77) | 6.28(2.36, 16.73) | 17.75 | 2.65(1.38) | 6.28(2.76) |
| suspected covid-19 | 3 | 6.21(2, 19.27) | 6.2(1.99, 19.32) | 13.08 | 2.63(1.22) | 6.2(2.4) |
| lymphocyte count decreased | 8 | 5.73(2.86, 11.47) | 5.72(2.88, 11.36) | 31.14 | 2.51(1.57) | 5.72(3.2) |
| pemphigoid | 3 | 5.69(1.83, 17.66) | 5.69(1.83, 17.73) | 11.58 | 2.51(1.09) | 5.68(2.2) |
| cholecystitis | 3 | 5.61(1.81, 17.42) | 5.61(1.8, 17.49) | 11.36 | 2.49(1.07) | 5.61(2.17) |
| bronchopulmonary aspergillosis | 3 | 5.57(1.8, 17.29) | 5.57(1.79, 17.36) | 11.23 | 2.48(1.06) | 5.56(2.16) |
| rash macular | 12 | 5.46(3.1, 9.62) | 5.44(3.08, 9.6) | 43.5 | 2.44(1.66) | 5.44(3.38) |
| skin infection | 4 | 5.37(2.02, 14.33) | 5.37(2.02, 14.31) | 14.21 | 2.42(1.16) | 5.37(2.36) |
| chills | 35 | 5.36(3.84, 7.48) | 5.32(3.81, 7.42) | 122.87 | 2.41(1.94) | 5.32(4.02) |
| skin exfoliation | 33 | 5.36(3.81, 7.55) | 5.32(3.81, 7.42) | 115.91 | 2.41(1.92) | 5.32(3.99) |
| cholestasis | 6 | 5.19(2.33, 11.57) | 5.19(2.32, 11.59) | 20.27 | 2.37(1.3) | 5.18(2.65) |
| photosensitivity reaction | 5 | 5.18(2.15, 12.46) | 5.17(2.14, 12.49) | 16.83 | 2.37(1.21) | 5.17(2.48) |
| generalised oedema | 3 | 5.18(1.67, 16.08) | 5.18(1.66, 16.14) | 10.1 | 2.37(0.96) | 5.17(2.01) |
| mass | 4 | 4.77(1.79, 12.72) | 4.77(1.79, 12.71) | 11.9 | 2.25(0.99) | 4.76(2.1) |
| therapy non-responder | 17 | 4.63(2.87, 7.45) | 4.61(2.88, 7.38) | 48.04 | 2.2(1.53) | 4.61(3.09) |
| neutrophil count decreased | 12 | 4.44(2.52, 7.83) | 4.43(2.51, 7.82) | 31.89 | 2.15(1.36) | 4.43(2.76) |
| melaena | 5 | 4.18(1.74, 10.04) | 4.17(1.73, 10.07) | 12.05 | 2.06(0.9) | 4.17(2) |
| mucosal inflammation | 6 | 4.05(1.82, 9.02) | 4.04(1.81, 9.02) | 13.73 | 2.01(0.94) | 4.04(2.07) |
| stomatitis | 15 | 3.97(2.39, 6.59) | 3.96(2.38, 6.59) | 33.17 | 1.98(1.28) | 3.96(2.59) |
| erythema | 52 | 3.76(2.86, 4.95) | 3.72(2.83, 4.89) | 103.88 | 1.9(1.5) | 3.72(2.96) |
| hepatic function abnormal | 8 | 3.75(1.87, 7.5) | 3.74(1.88, 7.43) | 16.08 | 1.9(0.96) | 3.74(2.09) |
| pyrexia | 73 | 3.71(2.95, 4.68) | 3.66(2.89, 4.63) | 141.74 | 1.87(1.54) | 3.66(3.01) |
| blister | 12 | 3.7(2.1, 6.52) | 3.69(2.09, 6.51) | 23.54 | 1.88(1.1) | 3.69(2.3) |
| sepsis | 22 | 3.53(2.32, 5.36) | 3.51(2.33, 5.3) | 39.55 | 1.81(1.22) | 3.51(2.47) |
| alanine aminotransferase increased | 10 | 3.37(1.81, 6.28) | 3.37(1.8, 6.31) | 16.64 | 1.75(0.9) | 3.37(2) |
| pruritus | 74 | 3.35(2.66, 4.22) | 3.3(2.61, 4.18) | 119.53 | 1.72(1.39) | 3.3(2.72) |
| white blood cell count decreased | 24 | 3.35(2.24, 5.01) | 3.34(2.26, 4.94) | 39.35 | 1.74(1.17) | 3.34(2.38) |
